# Residual Retrosternal Goiter and Thymolipoma After Cervical Thyroid Resection

**DOI:** 10.7759/cureus.71627

**Published:** 2024-10-16

**Authors:** Georgi Yankov, Magdalena Alexieva, Evgeni V Mekov

**Affiliations:** 1 Department of Thoracic Surgery, University Hospital “St. Ivan Rilski,” Medical University of Sofia, Sofia, BGR; 2 Department of Pulmonary Diseases, Medical University of Sofia, Sofia, BGR

**Keywords:** cervical thyroidectomy, recurrent goiter, retrosternal goiter, sternotomy, thymolipoma

## Abstract

Retrosternal goiters (RGs) are thyroid enlargements that extend into the mediastinum, representing 1%-20% of all goiters. While typically benign, their anatomical location can lead to significant clinical symptoms due to the compression of surrounding structures such as the trachea, esophagus, and major vessels. Surgical resection is the preferred treatment, particularly in symptomatic cases or when malignancy is suspected. In rare cases, RGs may co-occur with other mediastinal tumors, such as thymolipomas, complicating both diagnosis and management. We present a 39-year-old female with a residual retrosternal goiter after previous insufficient resection only of the cervical thyroid mass, leaving the mediastinal part in place. The patient underwent a total median sternotomy, and the retrosternal goiter, along with a concomitant thymolipoma, was successfully extirpated. Postoperative recovery was uneventful, and the patient remains in excellent condition at a seven-month follow-up.

## Introduction

Retrosternal goiters (RGs), also referred to as substernal or intrathoracic goiters, are thyroid enlargements that extend into the mediastinum, beyond the thoracic inlet [1]. RGs represent 1%-20% of all goiters, and although they are typically benign, their location within the mediastinum can lead to significant clinical symptoms due to the compression of surrounding structures, including the trachea, esophagus, and major blood vessels [2]. A giant retrosternal goiter (RG) is a rare disease with important medical significance due to its complexity in diagnostics and surgery.

In rare instances, RGs can co-occur with other mediastinal masses, such as thymolipomas [3]. Thymolipomas are slow-growing, benign tumors composed of both thymic and adipose tissues. They are often asymptomatic and discovered incidentally during imaging or surgery for other conditions. The combination of a retrosternal goiter with thymolipoma, as described in the current case, is extremely rare, with only a few cases reported in the literature.

This report presents a unique case of a 39-year-old female with residual retrosternal goiter and thymolipoma, following incomplete thyroid resection. The challenges in surgical management, particularly the need for a total median sternotomy, are discussed, along with a review of the relevant literature on surgical approaches and complications associated with RGs.

## Case presentation

**Figure 1:**
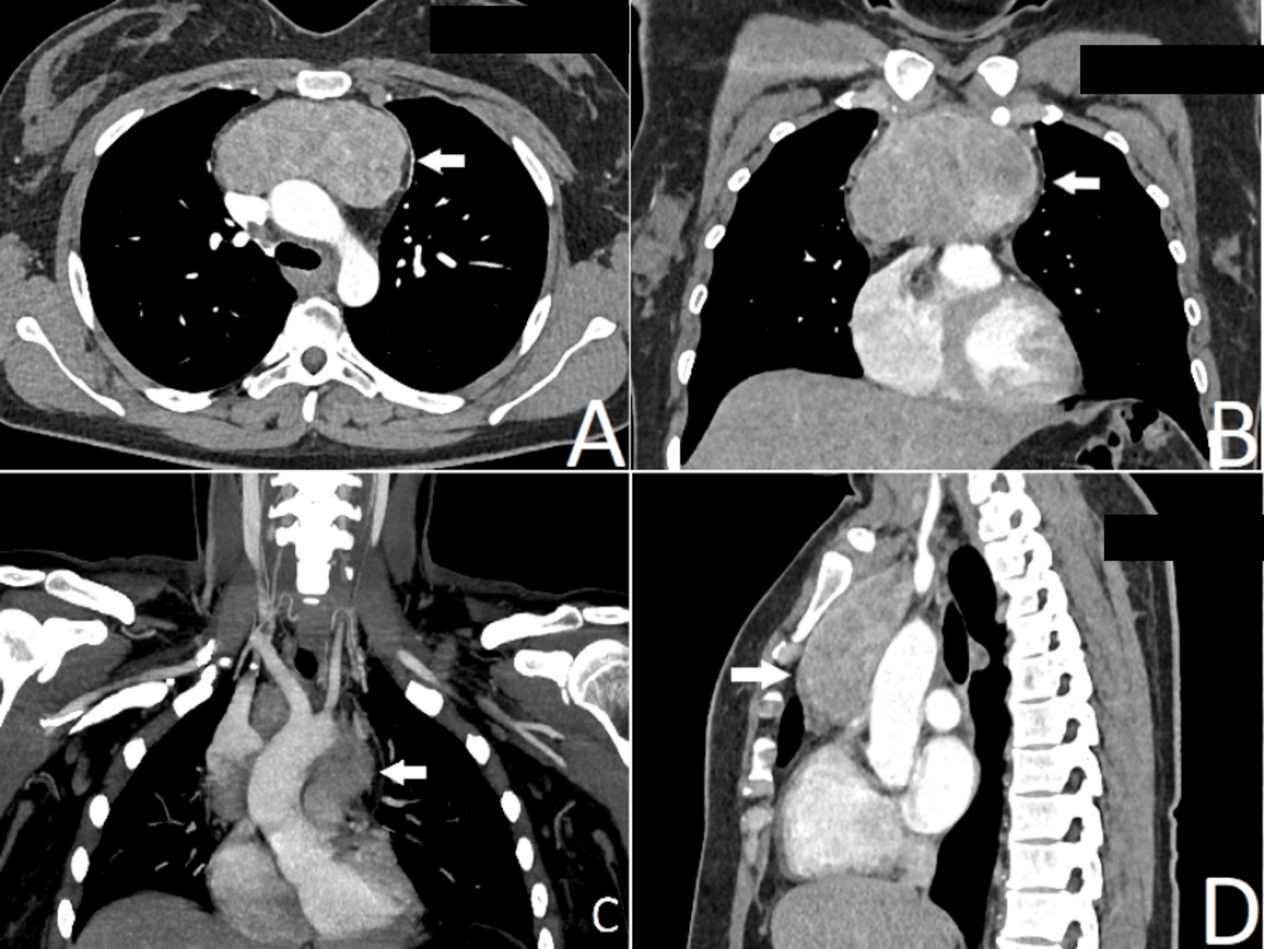
Contrasted computed tomography, showing a retrosternal goiter (white arrows).

A 39-year-old female was admitted to the thoracic surgery department with complaints of shortness of breath at rest, fatigue, chest pressure, and hoarseness for about a year. The patient had a medical history of total resection of the cervical thyroid gland due to multinodular goiter in another institution a year ago. During the first surgery, it was noted that the gland descended retrosternally in the thorax, but only the resection of the neck part through a Kocher incision was performed. She had no past or concomitant diseases. Laboratory tests along with thyroid hormones were in the normal range. Computed tomography (CT) scan visualized a well-demarked heterodense lesion in the anterior mediastinum 90/50 mm in the axial plane and 70 mm in the sagittal plane (Figure 1). There was no enlargement compared to the CT scan one year ago. The mass contrasted actively after the application of a contrast medium. It lay close to the superior vena cava, both brachiocephalic veins, ascending aorta, and aortic arch, as well as to the truncus pulmonalis, preserving the fat plane. The superior vena cava was partially compressed. A tru-cut biopsy proved residual RG in the superior anterior mediastinum.

**Figure 2:**
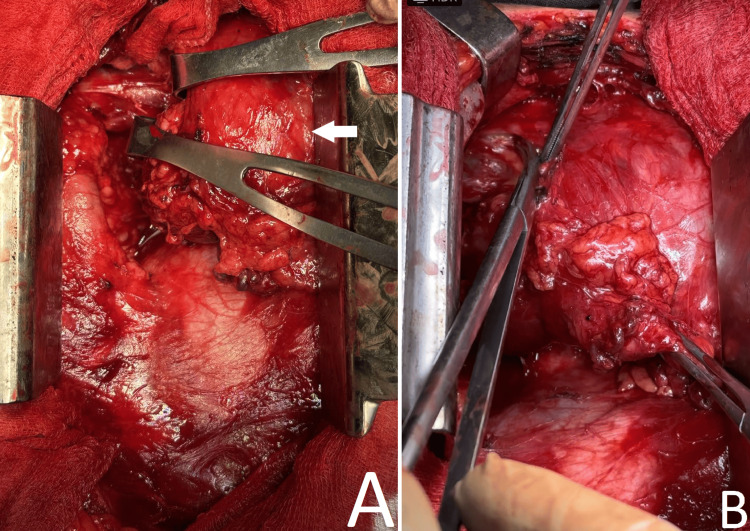
Intraoperative images of the retrosternal part of a cervico-mediastinal goiter (white arrow).

A total midline sternotomy was performed. An anterior-mediastinal residual thyroid lesion with a dimension of about 110/120 mm was found (Figure 2). The latter was fixed with tight adhesions to the neck, anterior tracheal wall, and mediastinal pleurae. Compression on the superior vena cava and the left brachiocephalic vein was exerted. The lesion was located anterior to the ascending aorta, arch, brachiocephalic trunk, and anterior pericardium and extended laterally to both mediastinal pleurae. Massive adhesions to the surrounding mediastinal tissues were present. By careful sharp dissection with scissors and harmonic device, the RG together with the surrounding perithymic adipose tissue was mobilized, freed, and extirpated. One retrosternal drainage was placed in the anterior mediastinum, and sternal osteosynthesis was carried out using wires.

**Figure 3:**
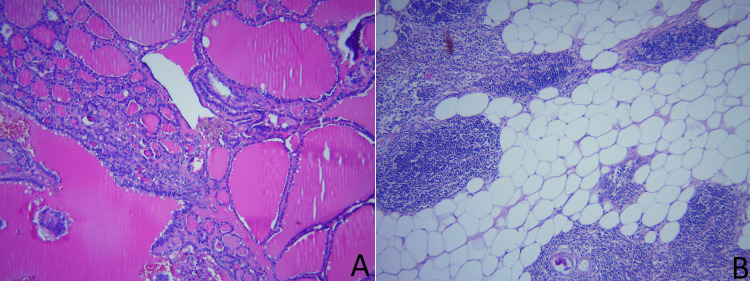
Histopathological image of the cervico-mediastinal goiter and thymolipoma. Panel A: Section showing multinodular goiter with thyroid follicles of varying sizes. The colloid-filled follicles are surrounded by a thin layer of cuboidal epithelium. Scale bar = 100 µm. Panel B: Histological section showing thymolipoma with a mix of adipose tissue and thymic lymphoid tissue. Scale bar = 100 µm. Magnification: 100×.

The histopathological result showed diffuse multinodular goiter combined with a thymolipoma (Figure 3).

The patient was discharged on the 10th postoperative day without any complications. At seven-month follow-up, she is in excellent overall condition.

## Discussion

This manuscript presents a 39-year-old female with a residual retrosternal goiter after previous insufficient resection only of the cervical thyroid mass, leaving the mediastinal part in place. A total midline sternotomy and the extirpation of the retrosternal goiter with thyroid tumor were performed.

About 16% of the patients in one study presented with a recurrent goiter following a previous operation most commonly due to inadvertent laceration, abandoning a thyroid in the thorax [4]. According to the medical records during the first surgery for cervical goiter through a neck collar incision, they incidentally found a deeply descending mediastinal part. Preoperative CT of the thorax was not performed, but only an ultrasound of the neck was carried out. They decided to remove only the cervical part of the thyroid gland and closed the cervicotomy. The lack of a thoracic surgeon led to the incomplete resection only of the cervical thyroid, leaving the retrosternal mediastinal part. In some cases, this could result in profuse mediastinal bleeding due to the proximal thyroid traction or from the remaining mediastinal gland.

The prevalence of intrathoracic goiter among patients with thyroidectomy is 1%-30% [2]. Indications for surgery are symptomatic RGs, fast enlargement, the risk of malignancy, the prevention of future complications such as the compression of the trachea or superior vena cava, Grave’s disease, and compromised aesthetic appearance.

The surgical approaches for the resection of RG are cervical and extracervical (thoracotomy, sternotomy, video-assisted thoracoscopic surgery, and robotic-assisted thoracoscopic surgery). The cervical approach is the most common approach for RG (84%), with the remainder necessitating total sternotomy (6.6%), thoracotomy (4%), or manubriotomy (3.1%) [5]. Huins et al. offer a three-level classification system for RGs and the corresponding operative approaches: 1) cervicotomy when located above the aortic arch (Th4), 2) manubriotomy when located from the aortic arch to the pericardium, and 3) total sternotomy when located below the right atrium [5]. We performed a total midline sternotomy due to the localization of the RG in the superior and anterior mediastinum and possible adhesions from the first surgery. We found in the literature only one similar case of a 59-year-old male with a giant recurrent intrathoracic goiter where only the cervical part was resected 13 years ago [6]. Clamshell thoracotomy, reverse sternotomy, and completion thyroidectomy were performed. However, there was no evidence of other thyroid pathology (e.g., thymolipoma) as in our case. An extremely rare case of a simultaneous appearance of thyrolipoma and thymolipoma in association with myasthenia gravis was described [3]. Thyrolipoma is a rare benign tumor of the thyroid gland composed of mature adipose tissue and thyroid tissue. Such cases have presented diagnostic challenges due to the similar mediastinal locations of these tumors.

Most RGs in one study were benign (93.4%), and only 6.6% had papillary thyroid carcinoma [4]. Another study showed a lower rate of multinodular goiter (58.5%), as the other pathological findings were papillary carcinoma (22.9%), medullary carcinoma (7.1%), anaplastic carcinoma (5.7%), thyroid lymphoma (5.7%), and thyroid adenoma (1.4%) [7].

Postsurgical outcomes for retrosternal goiters, especially when complicated by additional mediastinal masses such as thymolipomas, are generally favorable when complete resection is achieved. In our case, we did not observe any complications. However, the complication rate is higher in the extracervical approach compared to the cervical approach. The reported postoperative complications included postoperative hypocalcemia (3.4%), transient recurrent laryngeal nerve damage (2.6%), postoperative bleeding (0.75%), and acute respiratory failure with fatal outcomes caused by tracheomalacia (0.37%) [8]. The gold standard in the prevention of recurrent laryngeal nerve injury is its visualization, as intraoperative nerve monitoring is also very helpful. The prevalence of tracheomalacia (1%), superior vena cava syndrome (3.2%), and the intrathoracic approach rise more than 10-fold when RGs extend to the aortic arch [5].

## Conclusions

The combination of RG and thymolipoma is extremely rare. The management of residual thyroid masses is entirely surgical and challenging.
